# Improving a Mother to Child HIV Transmission Programme through Health System Redesign: Quality Improvement, Protocol Adjustment and Resource Addition

**DOI:** 10.1371/journal.pone.0013891

**Published:** 2010-11-09

**Authors:** Michele S. Youngleson, Paul Nkurunziza, Karen Jennings, Juanita Arendse, Kedar S. Mate, Pierre Barker

**Affiliations:** 1 Institute for Healthcare Improvement, Cambridge, Massachusetts, United States of America; 2 City Health Department, Cape Town, South Africa; 3 Department of Health, Western Cape Province, South Africa; 4 Department of Medicine, Weill-Cornell Medical College, New York, New York, United States of America; 5 Department of Pediatrics, University of North Carolina at Chapel Hill, Chapel Hill, North Carolina, United States of America; University of Stellenbosch, South Africa

## Abstract

**Background:**

Health systems that deliver prevention of mother to child transmission (PMTCT) services in low and middle income countries continue to underperform, resulting in thousands of unnecessary HIV infections of newborns each year. We used a combination of approaches to health systems strengthening to reduce transmission of HIV from mother to infant in a multi-facility public health system in South Africa.

**Methodology/Principal Findings:**

All primary care sites and specialized birthing centers in a resource constrained sub-district of Cape Metro District, South Africa, were enrolled in a quality improvement (QI) programme. All pregnant women receiving antenatal, intrapartum and postnatal infant care in the sub-district between January 2006 and March 2009 were included in the intervention that had a prototype-innovation phase and a rapid spread phase. System changes were introduced to help frontline healthcare workers to identify and improve performance gaps at each step of the PMTCT pathway. Improvement was facilitated and spread through the use of a Breakthrough Series Collaborative that accelerated learning and the spread of successful changes. Protocol changes and additional resources were introduced by provincial and municipal government. The proportion of HIV-exposed infants testing positive declined from 7.6% to 5%. Key intermediate PMTCT processes improved (antenatal AZT increased from 74% to 86%, PMTCT clients on HAART at the time of labour increased from 10% to 25%, intrapartum AZT increased from 43% to 84%, and postnatal HIV testing from 79% to 95%) compared to baseline.

**Conclusions/Significance:**

System improvement methods, protocol changes and addition/reallocation of resources contributed to improved PMTCT processes and outcomes in a resource constrained setting. The intervention requires a clear design, leadership buy-in, building local capacity to use systems improvement methods, and a reliable data system. A systems improvement approach offers a much needed approach to rapidly improve under-performing PMTCT implementation programmes at scale in sub-Saharan Africa.

## Introduction

More than twenty years into the HIV epidemic, infants in many part of the developing world continue to be infected during pregnancy, birth and infancy at unacceptably high rates. Application of standard anti-retroviral (ARV) protocols in high-income nations has, for the past decade, made maternal to child transmission of HIV a relatively rare event [1]–[3]. This achievement contrasts with results in sub-Saharan countries where, each year, nearly 400,000 infants continue to acquire HIV infection through perinatal HIV transmission, a consequence of limited drug regimens, poor access to care and poor service delivery [4].

Since 2002, the South African government has provided single-dose Nevirapine (sdNVP) prophylaxis to pregnant women in the public health system and highly active antiretroviral therapy (HAART) was made available from 2004 for pregnant women with low CD4 counts (<200 cell/dl). Dual ARV prophylaxis, Azidothymidine (AZT) and Nevirapine (NVP), was introduced in the Western Cape Province in 2004 with subsequent rollout to the remainder of the country in 2008 [5], [6]. Effectiveness of the South African program has been variable. In 2007, despite availability of sdNVP prophylaxis in 95% of antenatal clinics, a mother-to-child HIV transmission rate of 20.6% was reported in Kwa-Zulu Natal province nearly twice as high as would be expected from an effective Nevirapine-based prevention of mother-to-child transmission (PMTCT) programme [7]. After introduction of dual ARV prophylaxis in the same Province, peri-natal HIV transmission rates remained greater than 7%, still more than three times as high as would be expected from an effective dual ARV prophylaxis program [8], [9].

Shortly after dual ARV prophylaxis was introduced in the Western Cape, transmission rates of 8.8% were reported, decreasing to less than 5% by 2007 [10]. However, these successes hid substantial variation in outcomes within the Province. Between the different subdistricts in the Cape Metro District, for example, MTCT rates ranged between 3.6% to 7.5% in 2007 [6]. In the sub-district with the highest MTCT rate, Eastern sub-district (ESD), we applied a systems improvement approach to enhance performance of the PMTCT programme. Here we describe the components of the intervention, and the resulting improvements in reliability of care and reductions in perinatal HIV transmission rates.

## Materials and Methods

### Ethics

The ethics of undertaking the systems improvement intervention and analysis and dissemination of the findings were evaluated by the Office of Human Research Ethics of the University of North Carolina at Chapel Hill. The Office determined that, given the nature of the intervention and the de-identified characteristics of the data, the study was exempted from further IRB review. Written consent was not obtained from individual patients as this was a population based health systems intervention to improve existing guidelines and protocols, and applied to the patient population without exception or exclusion.

### Population studied

The ESD population (∼360,000) includes urban, peri-urban and rural communities. The birth rate is 20/1000 per annum (∼7,200/yr), and antenatal HIV prevalence is 17.4% (∼1,250 HIV exposed infants annually) [11]. The in-facility delivery rate in the district is high (92.9% in 2006 and 99.9% in 2008). 94% of HIV positive women on the PMTCT programme elected to exclusively formula feed from birth [12].

The majority of people living in ESD receive their medical care in the public facilities. In 2006, eight of the 15 primary care clinics offered antenatal care and all offered PMTCT infant follow up including PCR testing. Obstetric services are delivered in two birthing units. Although a proportion of women living in ESD receive antenatal care and deliver in adjacent sub-districts, the majority of women access post-natal infant follow up in ESD.

### Routine PMTCT Monitoring and Evaluation

PMTCT data was routinely collected in registers at the facilities and collated each month on paper-based forms that were submitted to the sub-district office for entry into electronic collation tables. These tables have built-in validation formulae to flag if numerators exceed denominators. Completeness and consistency of the data is actively monitored.

Infant mortality data is compiled from death notifications by the Department of Home Affairs. During the period of the intervention there was a two-year delay before these data became available to the sub-district. Infant mortality data from 2006 were therefore only available for review in 2008. Maternal mortality data were not available for the sub-district during the study period.

### Health System Partnership

The health systems intervention was a partnership between the Western Cape Provincial Department of Health and Cape Town Municipality City Health departments and the Institute for Healthcare Improvement (IHI). Working closely with the health departments, IHI provided a systems improvement design and a quality improvement (QI) expert who introduced QI methods to the sub-district facilities, trained managers in QI methods and collaborated closely with senior health department officials on project design and execution.

### Project structure

The project was implemented in two phases: a prototype phase in a sub-section of the sub-district (seven primary care clinics and the two birthing units) that formed a self-contained inter-facility PMTCT care and referral system, followed by a spread phase in which the systems improvement approach were disseminated through all 17 PMTCT-linked health facilities in ESD. The health systems intervention had three concurrent components: a quality improvement framework, policy and protocol changes, and targeted resource additions.

### Quality Improvement methods

#### Breakthrough Series Collaborative

The improvement project was structured as a “Breakthrough Series (BTS) Collaborative”, a QI model that promotes change simultaneously across large parts of the system [13]. All healthcare facilities in the selected referring systems were linked into a learning network to accelerate peer-to-peer learning through setting common aims and goals, systematically improving the reliability of HIV care using QI methods, and sharing successful strategies for improving PMTCT. The improvement project was time limited (innovation phase of 21 months, and spread phase of 18 months). At six-monthly intervals, representatives from the clinical sites gathered at workshops (“Learning Sessions”) to learn how to use specific QI methods based on the Model for Improvement and the PDSA (Plan-Do-Study-Act) cycle [14]–[16]. These methods included setting aims, process mapping of the PMTCT care pathway, using routine data to identify of gaps in care, root cause analysis of these gaps, selection of change ideas to close specific gaps, and use of rapid-cycle change iterative methods to test improvement ideas.

Between these learning sessions, clinics formed multidisciplinary improvement teams that applied these QI skills at individual facilities. In addition, project staff provided on-site mentoring in the use of QI methods. The intensity of support decreased over time from fortnightly on-site mentoring in the prototype phase to a more leveraged approach through monthly sub-district meetings in the spread phase as managers became familiar with the methodology.

Facility-based improvement activities resulted in the development of local solutions to local problems. These solutions were spread to other sites through extension by the project staff, Department of Health (DOH) managers and through routine monthly meetings and the six-monthly BTS Collaborative Learning Sessions described above.

#### Development of a “package” of successful ideas to spread change

Successful changes developed in the prototype phase were complied into a “Change Package” for testing and adaption by other facilities as the project spread (Table 1). Successful change strategies included: maximizing the use of existing resources through strategic redeployment of staff and services, reducing duplication, improving information transfer, ‘bundling’ interventions to fully utilize each of the patient's visits, and developing patient-centered approaches such as minimizing the number of clinic visits, improving geographical access to care and introducing psychologically supportive changes to retain patients in care.

**Table 1:** Summary of Protocol Changes, Resource Additions and QI Process Improvements that led to documented improvement.

Phase of PMTCT Programme	Documented Improvement	Associated Protocol Change	Associated Resource Allocation	Associated Process Improvement
Antenatal care	Mothers attending ANC at less than 20 weeks improved from 18% to 33%	None	Multiple antenatal sites across primary care clinics in sub-district improves accessStrategic placement of nurse to high volume antenatal clinic to eliminate backlog and wait time for antenatal booking	All women asked date of last menstrual period, pregnancy test done if indicated and immediate antenatal booking if pregnant
	Percent of clients receiving AZT before the onset of labour increased from 72% to 89%	Gestational age for start of AZT lowered from 32 weeks to 28 weeks gestation		Improved early antenatal booking procedures as described above
	Percent of HIV positive pregnant women receiving HAART before onset of labour increased from 10% to 23%		HAART clinic at primary care site with large antenatal clinic and high HIV prevalence	Improved communication between ANC and ARV clinic, antenatal clients walked over to ARV room.‘Mother's Day’ : dedicated ARV clinic day each week for pregnant women needing HAART.Mother2mothers program gives support and education to women on PMTCT program
Labour ward care	Increased percent of PMTCT clients receiving dual ARV therapyNVP from 73% to 87%AZT from 42% to 84%			Labour Ward PMTCT checklist.Monthly Labour Ward PMTCT data review meeting at sub-district office.
Post natal follow-up	Number of babies entered into PMTCT register increased from 29 to 36 per month at a test site			Improved PMTCT information flow from labour ward by stapling ANC card to infant health card
	Percent of babies on receiving PCR test for HIVIncreased from 82% to 97%	Infant PCR testing brought forward from 14 to 6 weeks		Monthly data review of infant PCR testing at sub-district office.Active, timely follow up of missing babies and results.

### Policy Changes and Resource Inputs

In addition to QI methods, strategic additional resources and policy changes were introduced by the sub-district and province during the study period. The sub-district increased the number of antenatal clinics providing antenatal care through introducing Basic Antenatal Care (BANC) to two additional clinics using existing staff [17]. In an effort to increase local access to HAART, three new ARV service points were introduced into primary care facilities during the project, supplementing the two existing hospital-based ARV facilities. These new primary care ARV clinics used a QI approach to boost ARV capacity by redeploying existing staff from the subdistrict hospitals. Later these facilities received added staff from the DOH.

Two major changes to the PMTCT protocol were introduced by the DOH during the study period. The timing of the introduction of AZT prophylaxis during pregnancy was advanced from 32 weeks gestation to 28 weeks gestation, and the age of HIV testing of infants was decreased from 14 weeks to 6 weeks. Both protocol changes were introduced simultaneously with uptake occurring over a period of a few months in the early part of the spread phase of the project (early 2008).

### Project Execution

From January 2006 to March 2009, facility staff and managers from the different branches of the DOH participated in this project attending a total of six Learning Session workshops in the BTS Collaborative design. Between these workshops, district managers and program coordinators analyzed performance data which was then used to assist frontline staff to test change ideas. Improvement ideas from the “change package” were also actively spread at monthly sub-district staff meetings and by program coordinators during routine clinic visits. Senior managers were kept informed through monthly project reports. A steering committee of district and DOH managers guided the process, sanctioned the spread of changes, and intervened to removed obstacles to improving PMTCT that were identified by the project.

### Measurement and Statistical Methods

All data were obtained from routine monthly sub-district PMTCT data collected at antenatal, labour ward and infant follow up clinics and collated by the Municipal and Provincial DOH. Data measuring the performance of PMTCT processes of care and infant HIV infection rates were tracked over time. We used run chart and Shewhart control chart analysis (Chartrunner, Productivity-Quality Systems, Inc, and QI Charts, Scoville Associates, Chapel Hill, NC) to determine the effect of system changes on the processes and outcomes of PMTCT care as changes were made over time [18]. To determine whether the improvements in performance were statistically significant, we analyzed data variation and trends, using a time series analysis [19]–[21]. We used categorical analysis (chi-square and unpaired t-test) to compare change in performance before and after the interventions, or between different sub-districts.

## Results

### Improvements to Antenatal Management of HIV-Infected Pregnant Women

Throughout the project, the early steps in the PMTCT pathway were already functioning well. Antenatal attendance (at least one visit) in the Western Cape remained over 97% throughout the study period [11]. HIV testing during pregnancy was also high: 98% at the beginning of the project and 99% by the end (Table 2). Steps in antenatal PMTCT prioritized for improvement included initial attendance at ANC *before* 20 weeks gestation, uptake of AZT prophylaxis, and referral for HAART.

**Table 2:** Summary of PMTCT process and outcome results.

Table of results				
	Care Process	Period before intervention	Period after intervention	Chi Squarep value
Antenatal Care	% pregnant women testing for HIV	98%(5340/5437)	99%(5346/5440)	p>0.05
	% first antenatal booking visit at <20 weeks gestation	18%(143/778)	33%(607/1830)	p<0.001
	Antenatal AZT	72%(558/776)	89%(1103/1258)	p<0.001
	Antenatal HAART	10%124/1243	25%122/486	P<0.001
Labour Ward	NVP	74%179/242	86%211/244	P<0.001
	AZT	43%105/242	84%206/244	p<0.001
Infant follow up	Percentage of infants on the PMTCT register receiving PCR (Eastern sub-district)	79%1677/2122	95%1553/1631	p<0.001
Outcomes	Perinatal HIV transmission rate in Eastern sub-district	7.6%(66/870)	5.0%(62/1248)	p = 0.013
	Perinatal HIV transmission rate in Cape Town Metro excluding Eastern sub-district	4.2%(247/5890)	3.8%(289/7657)	p = 0.22

#### Improving access to antenatal care

A number of changes improved early antenatal booking in the sub-district during the intervention period. Antenatal care was offered at a further two facilities using existing staff. At the sub-district's highest volume antenatal clinic (average 130 new ANC clients per month), early antenatal booking was increased through active case finding of pregnancies. All female patients attending the clinic for any reason were screened and tested for pregnancy, and referred for “same day” antenatal care. The backlog of clients waiting for the 1^st^ ANC visit was decreased by re-allocating a nurse practitioner to the antenatal clinic from within the existing facility staff pool. These changes resulted in a significant improvement in the percentage of pregnant women booking for antenatal care at less than 20 weeks gestation from 18% before the intervention to 33% following the interventions (p<0.001, Table 2).

#### Improving access to AZT at ANC

**Figure 1:**
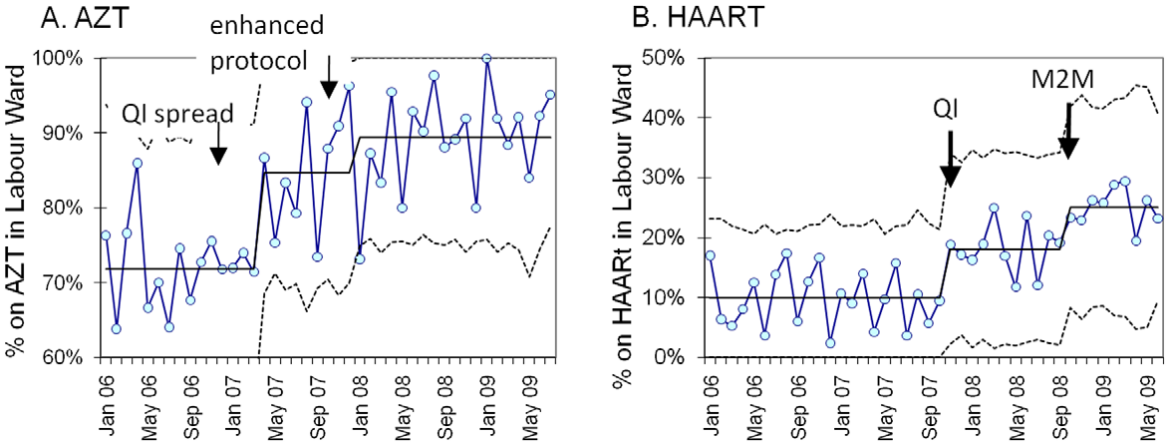
Percentage of HIV+ mothers receiving AZT before labour (A); and on HAART at time of delivery (B). Legend: Statistical process control p-charts showing changes (p<0.001) in mean percentage (with UCL and LCL) of eligible (HIV+) mothers (A) receiving AZT >2 weeks before labour (provincial protocol) and (B) on HAART at time of delivery in labour ward (p<0.001) showing effects of QI spread, protocol change and mother2mothers (M2M) interventions.

Further changes were tested to improve the uptake of AZT during the last trimester of pregnancy including: monthly review of AZT uptake indicators by sub-district managers (allowing poorly performing antenatal clinics to be identified and targeted management support to be allocated), and implementation of a new protocol to initiate AZT at 28 rather than 34 weeks gestation. These changes resulted in a significant stepwise improvement in baseline AZT uptake from 72% to 89% (p<0.001, Table 2, Figure 1A).

#### Improving access to HAART at ANC

As routine monitoring data did not capture maternal CD4 counts, we were not able to assess the proportion of HIV positive pregnant women eligible for HAART at the antenatal clinic. However, we were able to assess access to HAART by the time of delivery by measuring the number of HIV positive women on HAART at the time of delivery. Changes to the HAART referral process resulted in a significant improvement in the proportion of women who had started HAART by the time of delivery from 10% to 25% (p<0.001, Table 2, Figure 1B). The introduction of HAART initiation services in three primary care clinics with high volume ANC services contributed significantly to rapid and reliable access to ARV treatment for the ANC population. In these facilities, ANC clinic staff accompanied patients with low CD4 counts to the HAART clinic and transferred them directly to the ARV staff. In the facility with the largest ANC service, a weekly “‘Mother’s Day” dedicated to care of pregnant women was started at the HAART clinic within that site, and the Mothers2Mothers programme was introduced to offer of peer support and PMTCT education [22]. Strong referral systems between ANCs and ARV clinics were fostered by the Breakthrough Series Collaborative model which created a learning network of all facilities in the District. The number of new patients arriving at the HAART clinics monthly was monitored to detect drops in attendance. In these instances, local solutions for recalling patients were developed.

### Improvements in performance of PMTCT programme in the labour ward

**Figure 2:**
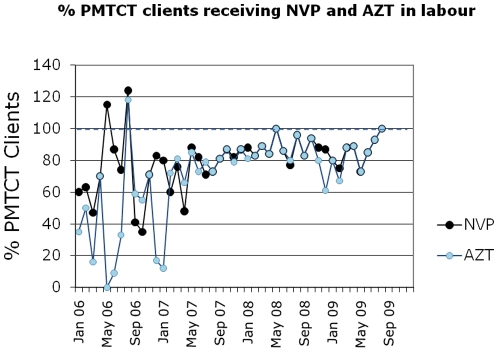
Percentage of HIV positive women receiving intrapartum administration of NVP and AZT. Legend: The quality of the data, and the reliability and variation of care in the administration of PMTCT in labour improved after the QI approach was introduced to the labour ward.

From 2006 to 2008, the proportion of eligible HIV positive women receiving dual ARV prophylaxis in the hospital labour ward improved for both NVP (74% to 86%, p<0.001, Table 2) and AZT (43% to 84%, p<0.001, Table 2, Figure 2). In addition, we observed a marked improvement in data quality during this time (reduction in monthly variation of data and elimination of data points above 100%). These improvements were associated with two systems innovations: a PMTCT Labour Ward Checklist (Figure S1) that prompted and documented care activities in the labour ward, and detailed monthly review of labour ward PMTCT data from the two obstetric sites in the sub-district by sub-district management. Identification of gaps in care and immediate feedback by the sub-district office to the labour wards allowed for a timely response to any performance failures. This process of data collection and routine sub-district monthly review has become a reliable tool for active programme monitoring and patient management. During the study period, it was noted that 10% of mothers on the PMTCT programme arrived at the two labour wards in advanced labour or after giving birth (too late to receive intrapartum ARVs). This problem may occur in similar health systems where NVP is dispensed only in the labour ward and not also at the antenatal clinic, blunting the effectiveness of the labour ward systems improvements described above.

### Improvement in follow-up of HIV-exposed infants

All primary care clinics in the Western Cape province offer infant PCR testing. More reliable data transfer between the labour ward and infant-testing sites in the subdistrict was achieved. Traditionally, PMTCT information was conveyed from labour ward to the primary care clinic by means of a stamp on the “Road To Health Card” issued at discharge from the labour ward. At the beginning of the project, a survey found that only 4% of PMTCT stamps were present or interpretable. Stapling the mother's antenatal patient record to the baby's Road To Health Card on discharge from the labour ward improved the data transfer to 80% because the Western Cape antenatal card clearly indicates maternal HIV status. This simple change increased the number of babies recorded in a test facility's cohort register by 24% (from an average of 29 HIV exposed babies per month to 36 per month).

**Figure 3:**
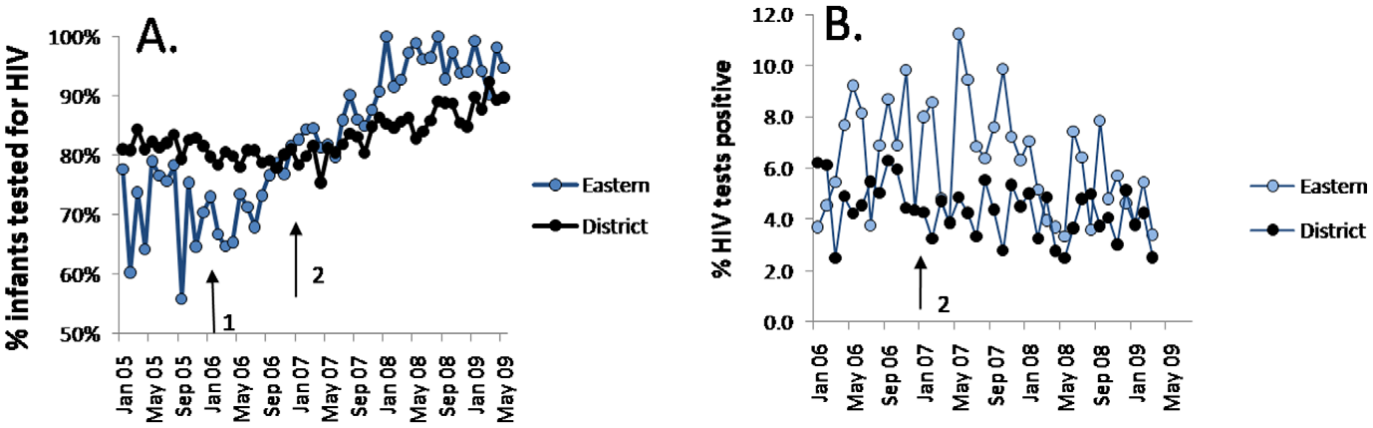
Percentage of infants tested for HIV (A) and percentage of infants testing HIV+ (B). Legend: A. Percent of HIV-exposed infants tested for HIV in Eastern sub-district (Eastern) vs. remainder of the Metro District (District) B. Percent of HIV-positive infants in Eastern sub-district vs remainder of Cape Town Metro District . “1” = start of the innovation phase, and “2” = start of spread phase.

During the study period, PCR testing of infants on the PMTCT register in ESD improved from 79% to 95% (p<0.001, Table 2). While PCR testing improved across the entire District during the study period, ESD improved at a faster rate and the proportion of babies receiving HIV PCR tests was significantly higher in Eastern (96%) than in the rest of the city (86.7%) (1,558 tested of 1,629 exposed infants versus 9,451 tested of 10,896 exposed infants, Figure 3A., p< 0.001).

We attribute the improvement in infant HIV testing rates to the introduction of a rapid response system to track infants who had not tested through a monthly PMTCT data review meeting at the sub-district office, and a protocol change in November 2007 to provide HIV PCR testing for infants at 6 weeks (coinciding with the 1^st^ immunization visit) rather than 14 weeks.

### Outcomes

Comparing the 12 months before the spread phase of the intervention (from November 2006) with the last 12 months of the intervention (ending March 2009), the HIV positive rate for infants tested with PCR in Eastern subdistrict decreased significantly from 7.6% to 5.0% (p =  0.013, Table 2, Figure 3B). The decline in the HIV positive rate for infants tested with PCR in the remainder of the Cape Town Metro District (excluding Eastern sub-district) was not statistically significant during the same time period (declined from 4.2% to 3.8%, p = 0.22, Figure 3B). Despite the improvements in infant PCR positive rates in the Eastern sub-district, infant PCR positive rates remained higher in Eastern sub-district than the rest of the Cape Town Metro District (5% versus 3.8%, 62/1248 versus 289/7657, p = 0.044) at the end of the study period. Infant mortality data for the sub-district was not available at the time of this analysis so we were unable to track the impact of the intervention on infant survival.

## Discussion

This project shows that significant improvements in district-wide performance of PMTCT processes and outcomes can be achieved through a combination of quality improvement interventions, introduction of better PMTCT protocols and the strategic addition or reallocation of resources (details provided in Supplement 2). We report improvement in the primary outcome (the proportion of positive HIV PCR tests in infants), which dropped by a third to reach the annualized target of 5% in the sub-district for the first time. In addition, improvements in PMTCT processes occurred throughout the continuum of care from early antenatal booking to the postnatal visit at 6–14 weeks. For HIV-infected pregnant women, the ability of the local health system to deliver either AZT or HAART during the third trimester was significantly enhanced as were labour ward practices.

The adoption of new PMTCT protocols and strategic addition of resources contributed to these improvements. In addition, for some of the processes that we targeted, the intervention sub-district improved to a greater degree than any other sub-district in the region. Through a time-series analysis, we were able to infer a strong association between the timing and resultant improvements in PMTCT process performance.

Several factors contributed to the success of our intervention. The project was conducted in a high performing public health system that already had a culture of reflective data analysis, and was able to effectively introduce protocol changes and add strategic resources. The structure of the routine PMTCT monitoring system facilitated the use of data to drive improvement because it is linked to patient registers that are meaningful to frontline staff. The health system leadership used PMTCT process data feedback to encourage participation in District-wide learning opportunities, and supported the testing of new ideas and the spread of successful interventions. A responsive District leadership deployed additional strategic resources when needed.

In addition to these local “host” factors, the Breakthrough Series (BTS) Collaborative learning system used in this project was a central mechanism for engaging front line health care providers in local innovation that led to improvements and facilitated rapid diffusion of successful changes. While there is limited experience using similar learning networks in resource-poor settings [23]–[26], this structure has been deployed extensively in the developed world to improve disease management and health systems [27]–[29]. Published reports indicate that the BTS mechanism has been successful in accelerating and spreading changes within large healthcare organizations and improving outcomes on a national scale [29]. However, the effectiveness of the BTS model has been inconsistent, often due to variations in the design and execution of the model [28]–[30]. Our results suggest that a BTS-based approach can be effectively applied at a sub-district level to improve specific health system processes and outcomes.

As policy changes, improvement methods and resource additions were often deployed simultaneously, one important limitation of our analysis is that attribution of results to any one of these three approaches is difficult. We utilized an annotated time series analysis to correlate the introduction of specific changes with resulting PMTCT process performance shifts in order to help understand which changes resulted in the improvement described above. This analytic method remains one of the best available approaches to understanding the effects of an intervention in “real-life,” uncontrolled trial settings.

An important confounder in this study was the protocol change that moved the timing of infant PCR testing forward by eight weeks (from 14 weeks of age to 6 weeks). This change may have biased the outcome towards a lower transmission rate in breast-fed or mixed fed babies who would formerly have been exposed to eight additional weeks of breast milk by the time of PCR testing. Although labour ward data indicate that 94% of women in our cohort elected to exclusively formula feed from delivery, the impact of this change will depend on the prevalence of mixed feeding which we cannot estimate from available data.

The likely adoption of more recent WHO guidelines that promote long-term neviripine prophylaxis for breast fed infants is likely to reduce the percentage of HIV positive mothers electing to formula feed [31].

The effects of improved PMTCT policies and protocols on reducing perinatal HIV transmission rates have been reported around the world [32]–[34]. Recent major policy changes in the South African PMTCT policy (introduction of dual ARV prophylaxis, early antenatal AZT, increase of CD4 threshold for HAART initiation in pregnancy from 200 to 350 cells/dl), clear government-led national targets (e.g., reduction of perinatal HIV transmission to <5%), ongoing ARV prophylaxis to infants exposed to breast milk, and improvements in PMTCT delivery are expected to reduce the high perinatal HIV transmission rates in South Africa [35]. However, variation in the ability of local health departments to deliver PMTCT interventions will result in major differences in PMTCT outcomes across the country. This regional variation in health system performance is mirrored in many other healthcare systems[36]. In many instances, even after verified research studies and consensus practice guidelines call for specific clinical practices, widespread implementation can be delayed by many years [37]; the existence of strong guidelines and trained staff does not guarantee successful programme implementation. Achieving the full potential of any protocol depends on the ability of the healthcare systems to deliver it to the right patients in a timely fashion [38]. This study highlights the role that a health systems improvement methodology can play in supporting healthcare workers to overcome systems barriers and deliver reliable care that maximizes the potential of existing protocols.

The comprehensive nature of this intervention, involving all facilities with PMTCT activities in the sub-district across the continuum of the PMTCT program, provides a model for how district-level activities can be coordinated to achieve improved results. We conclude that a health systems improvement approach will lead to major improvements in PMTCT processes performance and outcomes in a defined health region given certain conditions: mediation of improvement efforts through regular feedback of reliable performance data, a learning network model that connects all facilities in the area, a design that includes both an innovation (learning) and a spread phase, and close support from leaders who introduce protocol amendments and strategic resources when needed. The intervention described requires a clear design, leadership buy-in, local capacitation in systems improvement methods, and a reliable data system. The successful intervention to decrease HIV transmission clearly benefitted from the resources and leadership provided by the Western Cape Department of Health. Whilst further testing and adaptation of these systems principles to other less well-resourced settings in South Africa and elsewhere are much needed, a systems improvement approach offers a potential way to rapidly improve under-performing PMTCT implementation programmes in sub-Saharan Africa.

## Supporting Information

Figure S1 Labour Ward Checklist. Checklist tool that was developed to improve performance of PMTCT care processes in labour ward.
(0.05 MB TIF)
