# Supplementary figures and images for: Improving a Mother to Child HIV Transmission Programme through Health System Redesign: Quality Improvement, Protocol Adjustment and Resource Addition
Source: PLoS One. 2010 Nov 9;5(11):e13891. doi: 10.1371/journal.pone.0013891 (PMC2976693; doi:10.1371/journal.pone.0013891)

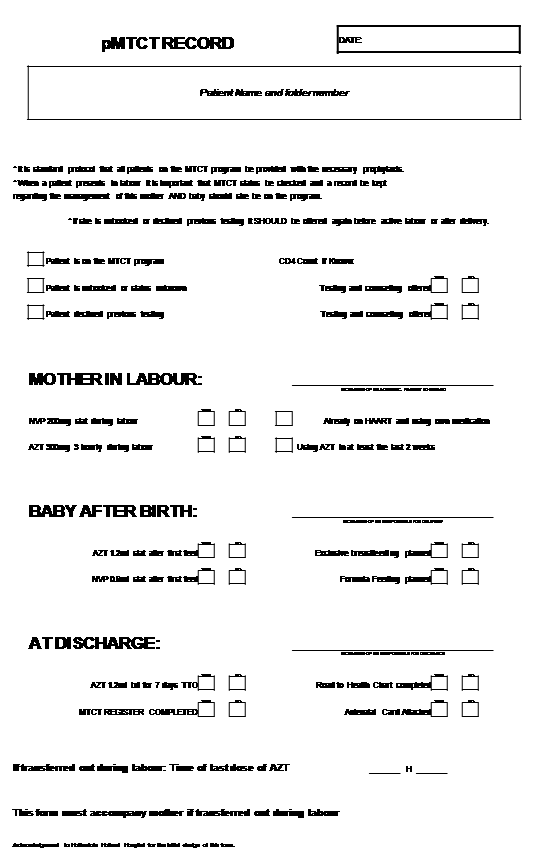

Supplement: Figure S1 — Labour Ward Checklist. Checklist tool that was developed to improve performance of PMTCT care processes in labour ward. (0.05 MB TIF) [file pone.0013891.s001.tif]
